# New Books

**Published:** 2005-06

**Asbestos and Fire**

Rachel Maines

Piscataway, NJ:Rutgers University Press, 2005. 288 pp. ISBN: 0-8135-3575-1, $34.95

**Chemical Genomics: Reviews and Protocols**

Edward D. Zanders

Totowa, NJ:Humana Press, Inc., 2005. 300 pp. ISBN: 1-58829-399-8, $110

**Computational Genetics and Genomics: Tools for Understanding Disease**

Gary Peltz

Totowa, NJ:Humana Press, Inc., 2005. 286 pp. ISBN: 1-58829-187-1, $125

**Epigenetics**

Bruce Stillman, ed.

Woodbury, NY:Cold Spring Harbor Laboratory Press, 2005. 520 pp. ISBN: 0-87969-729-6, $295

**Essential Mathematics and Statistics for Science**

Graham Currell

Hoboken, NJ:John Wiley & Sons, Inc., 2005. 344 pp. ISBN: 0-470-02228-0, $115.50

**EU Environmental Law**

Maria Lee

Oxford, UK:Hart Publishing, 2005. 270 pp. ISBN: 1-84113-410-4, $50

**Handbook of Genome Research: Genomics, Proteomics, Metabolomics, Bioinformatics, Ethical and Legal Issues**

Christoph W. Sensen

Hoboken, NJ:John Wiley & Sons, Inc., 2005. 614 pp. ISBN: 3-527-31348-6, $355

**Health Effects of Transport-Related Air Pollution**

M. Krzyzanowski, B. Kuna-Dibbert, J. Schneider

Geneva:World Health Organization Press, 2005. 275 pp. ISBN: 92-890-1373-7, $54

**Microarrays in Clinical Diagnostics**

Thomas O. Joos, Paolo Fortina

Totowa, NJ:Humana Press, Inc., 2005. 300 pp. ISBN: 1-58829-394-7, $135

**National Environmental Accounting: Bridging the Gap Between Ecology and Economy**

Joy E. Hecht

Washington, DC:RFF Press, 2005. 240 pp. ISBN: 1-891853-93-7, $60

**Pharmacogenomics: Methods and Applications**

Federico Innocenti

Totowa, NJ:Humana Press, Inc., 2005. 250 pp. ISBN: 1-58829-440-4, $125

**Proteomics in Cancer Research**

Daniel C. Liebler, Emanuel F. Petricoin, Lance A. Liotta

Hoboken, NJ:John Wiley & Sons, Inc., 2005. 202 pp. ISBN: 0-471-44476-6, $150

**Rational Choice and Judgment: Decision Analysis for the Decider**

Rex Brown

Hoboken, NJ:John Wiley & Sons, Inc., 2005. 245 pp. ISBN: 0-471-20237-1, $79.95

**Sprawl Costs: Economic Impacts of Unchecked Development**

Robert Burchell, Anthony Downs, Sahan Mukherji

Washington, DC:Island Press, 2005. 224 pp. ISBN: 1-55963-530-4, $18.75

**Sustainable Energy: Choosing among Options**

Jefferson W. Tester, Elisabeth M. Drake, Michael J. Driscoll, Michael W. Golay, William A. Peters

Cambridge, MA:MIT Press, 2005. 872 pp. ISBN: 0-262-20153-4, $78

**The Evolution of the Genome**

T. Gregory

Burlington, MA:Elsevier, 2005. 768 pp. ISBN: 0-12-301463-8, $69.95

**The Oncogenomics Handbook**

William J. LaRochelle, Richard A. Shimkets

Totowa, NJ:Humana Press, Inc., 2005. 752 pp. ISBN: 1-58829-425-0, $195

**The Proteomics Protocols Handbook**

John M. Walker

Totowa, NJ:Humana Press, Inc., 2005. 1,016 pp. ISBN: 1-58829-343-2, $175

**The World Health Report 2005**

World Health Organization

Geneva, Switzerland:World Health Organization Press, 2005. 252 pp. ISBN: 92-4-156290-0, $36

**Water Quality Hazards and Dispersion of Pollutants**

Wlodzimierz Czernuszenko, Pawel Rowinski

New York:Springer-Verlag, 2005. 250 pp. ISBN: 0-387-23321-0, $139

